# Biological screening of a unique drug library targeting MRGPRX2

**DOI:** 10.3389/fimmu.2022.997389

**Published:** 2022-10-21

**Authors:** Fan Yang, Nathachit Limjunyawong, Qi Peng, John T. Schroeder, Sarbjit Saini, Donald MacGlashan, Xinzhong Dong, Li Gao

**Affiliations:** ^1^Division of Allergy and Clinical Immunology, The Johns Hopkins University School of Medicine, Baltimore, MD, United States; ^2^Department of Dermatology, Shengjing Hospital of China Medical University, Shenyang, China; ^3^The Solomon H. Snyder Department of Neuroscience, The Johns Hopkins University School of Medicine, Baltimore, MD, United States

**Keywords:** drug hypersensitivity reaction (DHR), high-throughput screening (HTS), G-protein coupled receptor (GPCR), MRGPRX2, mutation, tyrosine-kinase inhibitor (TKI)

## Abstract

**Background:**

Allergic drug reaction or drug allergy is an immunologically mediated drug hypersensitivity reaction (DHR). G-protein coupled receptors (GPCRs) are common drug targets and communicate extracellular signals that initiate cellular responses. Recent evidence shows that GPCR MRGPRX2 is of major importance in IgE-independent pseudo-allergic DHRs based on the suspected interactions between many FDA-approved peptidergic compounds and MRGPRX2.

**Objective:**

Our aim was to uncover novel MRGPRX2-selective and -potent agonists as drug candidates responsible for clinical features of pseudo-allergic DHRs.

**Methods:**

We conducted a primary high-throughput screening (HTS), coupled with mutagenesis targeting the MRGPRX2 N62S mutation, on a panel of 3,456 library compounds. We discovered pharmacologically active hit compounds as agonists of the MRGPRX2 protein according to high degrees of potency evaluated by the calcium response and validated by the degranulation assay. Using the molecular tool Forge, we also characterized the structure-activity relationship shared by identified hit compounds.

**Results:**

The alternative allele of single nucleotide polymorphism rs10833049 (N62S) in MRGPRX2 demonstrated *loss-of-function* property in response to substance P and antineoplastic agent daunorubicin hydrochloride. We applied a unique assay system targeting the N62S mutation to the HTS and identified 84 MRGPRX2-selective active hit compounds representing diverse classes according to primary drug indications. The top five highly represented groups included fluoroquinolone and non-fluoroquinolone antibiotics; antidepressive/antipsychotic; antihistaminic and antineoplastic agents. We classified hit compounds into 14 clusters representing a variety of chemical and drug classes beyond those reported, such as opioids, neuromuscular blocking agents, and fluoroquinolones. We further demonstrated MRGPRX2-dependent degranulation in the human mast cell line LAD2 cells induced by three novel agonists representing the non-fluoroquinolone antibiotics (bacitracin A), anti-allergic agents (brompheniramine maleate) and tyrosine-kinase inhibitors (imatinib mesylate).

**Conclusion:**

Our findings could facilitate the development of interventions for personalized prevention and treatment of DHRs, as well as future pharmacogenetic investigations of MRGPRX2 in relevant disease cohorts.

## Introduction

Allergic drug reactions, or immunologically mediated drug hypersensitivity reactions (DHRs), account for approximately 6% to 10% of all adverse drug reactions (1). In contrast, pseudo-allergic DHR is a type of non-immune-mediated drug hypersensitivity in which drugs directly activate the effector mechanism of inflammation (*i.e.*, direct mast cell activation and degranulation) without the involvement of adaptive immune mechanism (*i.e.*, drug-specific IgE) (2). Pseudo-allergic DHRs share clinical symptoms with anaphylactic reactions and are especially dangerous because they can occur upon the first exposure to a drug.

G-protein coupled receptors (GPCRs) are common drug targets and can modulate diverse signaling pathways, often in a ligand-specific manner (3). MRGPRX2 belongs to a new subfamily of GPCRs—the MAS-related GPCRs (MRGPRs) (4). MRGPRX2 activation mediates pseudo-allergic DHRs when interacting with certain peptidergic drugs (*e.g.*, icatibant) that potentially induce injection-site reactions (*e.g.*, erythema and swelling) (5). Several small-molecule drugs (such as neuromuscular blocking agents (NMBA) and fluoroquinolones) may also produce anaphylactic events through MRGPRX2 (5, 6). MRGPRX2 has attracted interest among the approximately 50 members of the MRGPR family because it mediates many pathological conditions related to host defense, drug-induced anaphylactoid reactions, neurogenic inflammation, pain, itch, and chronic inflammatory diseases (7).

Since the seminal discovery of MRGPRX2 as a significant mast cell (MC) receptor responsible for non-IgE-mediated MC activation, researchers have reported a plethora of endogenous and exogenous MRGPRX2 agonists (6, 8). These include basic secretagogues and neurokinins and commonly used small-molecule drugs as mentioned above. In the case of small-molecule drugs, MC degranulation occurs under *in vitro* experimental conditions, which may cause MRGPRX2-related systemic pseudo-allergic reactions, particularly acute urticaria and anaphylaxis (9, 10). Peptidergic drugs (*e.g.*, icatibant) that frequently induce injection-site reactions also cause MC degranulation in an MRGPRX2-dependent manner (5). Ligand stereochemistry studies indicate that opioids such as the dextro-enantiomers and N-methyl substituted scaffolds activate MRGPRX2 (11). Finally, at least 18 cationic amphiphilic drugs are agonists for MRGPRX2 (12). These drugs are commonly used as antidepressants, antipsychotics, anti-allergic agents, or antispasmodics. We speculate that a high-throughput screening (HTS) facilitated by exploration of the structure-activity relationship (SAR) may discover novel agonists associated with MRGPRX2-dependent MC activation and as potential candidates for pseudo-allergic DHRs.

MRGPRX2 is a protein-coding gene encompassing a 6.2kb region on human chromosome 11 with only two coding exons. We found six missense variants in MRGPRX2 with a minor allele frequency (MAF) ≥ 0.001 (rs11024970 (N16H), rs10833049 (N62S), rs118176470 (V108A), rs201846837 (M119I), rs150365137 (W243R) and rs117328742 (S313R)). Among these, two are common, with a MAF ≥ 0.05: N16H (0.11) and N62S (0.32) (13). Recent research confirmed N62S as a loss-of-function (LOF) mutation that is protective for ulcerative colitis, with decreased activation of mast cells (14). Thus, the disease-associated N62S mutation represents a plausible candidate to probe basic biological processes and facilitate pharmacogenetic studies targeting MRGPRX2. We hypothesized that MRGPRX2-mediated pseudo-allergic reactions might involve drug- (*e.g.*, the off-target pharmacological activity of certain drugs on MRGPRX2) or host- (*e.g.*, specific genetic polymorphisms of MRGPRX2) related factors. In this study, we developed a unique assay system and performed an *in vitro* biological HTS of the Johns Hopkins Drug Library (JHDL). We identified selective agonists for MRGPRX2 sharing distinct structural similarities. The ultimate goal was to develop new strategies to improve drug efficiency and safety and implement personalized therapy for DHRs.

## Materials and methods

### Generation of MRGPRX2 N62S mutant construct and sequence confirmation

**Figure 1:**
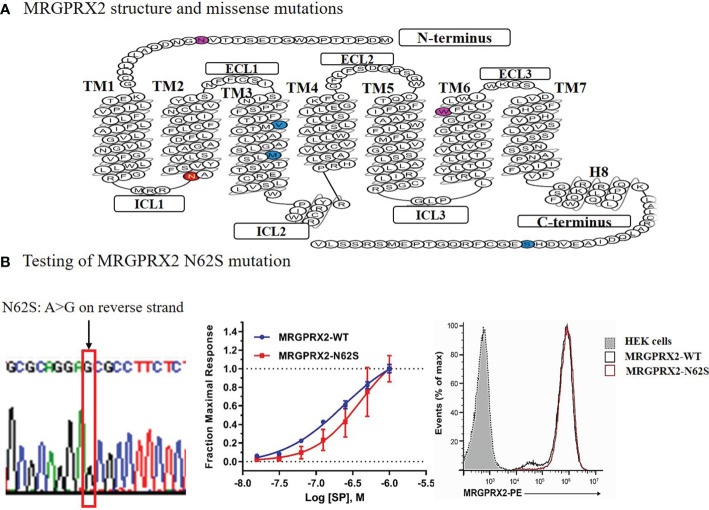
Diagram of the secondary structure of MRGPRX2 with missense mutations denoted **(A)** and functional testing of the N62S mutation in HEK293-Gα15 cells stably expressing the N62S mutation compared to the wild-type receptor **(B)**. The “*loss-of-function*” effects of N62S mutation on MRGPRX2 was confirmed by reduced cytosolic calcium responses (*middle*), despite similar levels of cell surface expression of the receptor measured by flow cytometry using an anti-MRGPRX2 antibody (*right*). We confirmed the construct containing the N62S mutation by Sanger sequencing (*left*).

We performed site-directed mutagenesis to generate a mutant construct expressing the selected MRGPRX2 mutation Asn62Ser or N62S (amino acid substitution from asparagine to serine at position 62). A mammalian expression construct of wild-type (WT) MRGPRX2 (MRGPRX2-WT) in pcDNA 3.1 vector was generated in Dr. Xinzhong Dong’s laboratory. We used this MRGPRX2-WT as backbones and used the Q5^®^ Site-Directed Mutagenesis Kit (New England Biolabs) to generate the mutant construct expressing MRGPRX2 variant N62S (a single base pair substitution, from A to G). We sequence-confirmed the constructs (**Figure 1B**, *left* panel).

### Making the stable cell lines expressing MRGPRX2 wild-type and mutant constructs

We cultured HEK293-Gα15 cells (HEK293 cells stably overexpressing G protein Gα15) on 24-well plates with a seeding density of 2.5x10^4^ cells/per well, cells were maintained at 37 °C in a humidified atmosphere containing 5% CO_2_. After 48 hours of culture incubation in Dulbecco’s Modified Eagle’s Medium (DMEM), pH 7.0 -7.6, supplemented with 10% FBS and 1% penicillin/streptomycin, we transfected the cells with plasmids encoding the WT or mutant receptors utilizing Lipofectamine™ 3000 Reagent (Invitrogen). We then selected clones stably expressing WT and mutant MRGPRX2 constructs by fluorescence-activated cell sorting (FACS), using a monoclonal antibody (BioLegend) against MRGPRX2. We selected clones with relatively high expression of MRGPRX2 compared to unstained cells. We followed this with a validation test of calcium release to a known MRGPRX2 ligand substance P (SP) eight weeks after selection (**Figure 1B**, *middle* panel). Each sample was run in triplicate and we repeated the experiments at least three times.

### Primary *in vitro* screen and selection of top candidate drugs

**Figure 2:**
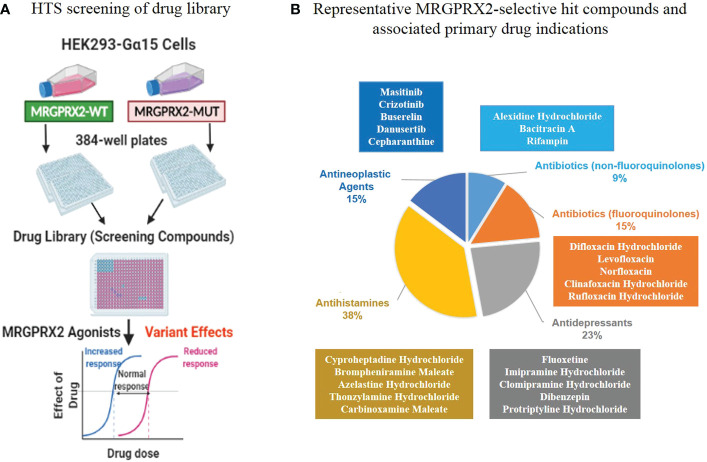
An *in vitro* biological high-throughput screening (HTS) of FDA-approved drug library for identifying agonists for MRGPRX2. **(A)** Workflow of primary HTS. **(B)** The top five major classes (according to the primary drug indications) containing most of the MRGPRX2-selective agonists are depicted along with representative hit compounds in each class (the top five).

We performed an experimental HTS on the Johns Hopkins Drug Library, which includes 1,811 (57%) FDA-approved drugs among 3,456 total compounds (28% of all known drugs worldwide) (15). We performed the HTS at the Johns Hopkins University ChemBioCORE Facility and employed HEK293-Gα15 cell lines that stably expressed MRGPRX2 protein. These cell lines included both the wild-type (MRGPRX2-WT) and mutant (MRGPRX2-MUT) targeting the N62S mutation (**Figure 2A**).

We utilized 10 μM compounds (each compound was tested in triplicate) and calculated the compound effect using the signal-to-background fluorescence ratio (*see* Supplementary Methods). We used phosphate-buffered saline (PBS) with 1% DMSO as a negative control and MRGPRX2 agonist SP (10 μM) in the same buffer served as a positive control on each testing plate. We further calculated B-scores, which provided an effective, non-controls-based methodology to deal with positional effects (16). If the compound caused more than three times the standard deviation (SD) of the B-scores of the library compounds, we considered the compound active as an agonist of the MRGPRX2 protein (17).

### Clustering analysis utilizing a molecular fingerprint tool for drug discovery

To identify highly similar molecular properties (*e.g.*, electrostatic fields, shape, or common motif) shared by MRGPRX2 agonists, we utilized the molecular tool Forge, which uses molecular alignments to make meaningful comparisons across chemical series (18). We selected the Morgan molecular fingerprint-based algorithm (*i.e.*, extended-connectivity fingerprint ECFP4) (19), which provided a clustering similarity score (*e.g.*, threshold 0.6) between the result molecule and the target molecule. The score is key in deciding the validity and potential activity of alignments and molecules.

### Generation of dose-response curves

As shown in **Supplementary Figure 1**, we selected anthracyclines (DNR-DOX), which are widely used in human cancer chemotherapy for validation, using the 384-well plate format. We applied eight concentrations on a logarithmic scale (-6.5, -6, -5.5, -5, -4.5, -4, -3.5, -3) with a top test concentration of 1 x 10^-3^ M or 1 mM for assessing each drug in triplicates and repeated the experiment independently. We plotted dose-response curves over an eight concentration-effect range using GraphPad Prism (version 7) and further calculated EC_50_ value to determine the potency of each testing drug.

### β-hexosaminidase release assay in human mast cells

The human mast cell line LAD2 (Laboratory of Allergic Diseases-2) was obtained from Dean Metcalfe, MD (National Institute of Allergy and Infectious Diseases, National Institutes of Health, Bethesda, MD). Three selected agonistic drugs were ordered from Sigma-Aldrich (bacitracin A), Fisher Scientific (brompheniramine maleate) and Selleck Chemicals (imatinib mesylate) in single vials (25 mg). We included two negative and two positive controls for the validation experiments in LAD2 cells. Drugs were tested for β-hexosaminidase release as an indicator of MC degranulation in the LAD2 cells and MRGPRX2-deficient LAD2 cells (as a negative control) generated by using CRISPR/Cas9, as we have described previously (5). Additionally, vehicle control (without any drugs) was also included as the negative control. Drugs were dissolved in PBS (vehicle) and six concentrations were tested for each drug; the known MRGPRX2 agonist C48/80 (10 μg/mL) and Tween 20 (1%), which is a known mast cell activator *via* MRGPRX2 independent mechanism, were used as positive controls. Two-way ANOVA with the Sidak multiple comparisons test was used for statistical analysis (n=6).

## Results

### MRGPRX2 N62S mutation demonstrated LOF property in response to substance P

As shown in **Figure 1A**, GPCR MRGPRX2 contains a common seven transmembrane (7-TM) architecture linked by three extracellular (ECL) and three intracellular (ICL) loops. Computational protein prediction program polymorphism phenotyping v2 (PolyPhen-2) predicted that N62S would be a damaging variant, with a score of 0.976 (in *red*) on a scale of 0 to 1 (0 is benign). The N16H and W243R variants’ scores were 0.937 and 0.760 (in *purple*), respectively. No predicted scores were available for the other three variants (in *blue*). Further, Phobius (a program that predicts transmembrane topology and signal peptides from the amino acid sequence of a protein) predicted that N16H would affect extracellular Domain 1, whereas N62S affects cytoplasmic Domain 1 of MRGPRX2 (20).

We have developed a novel screening system utilizing transfected HEK293-Gα15 cells with wild-type and mutant (N62S missense mutation) MRGPRX2. We found that the rise in intracellular Ca^2+^ in response to SP was significantly inhibited in HEK293-Gα15 cells that stably expressed the mutant N62S (**Figure 1B**, *middle* panel), compared to the WT receptor at various concentrations (*P*<0.01). This difference held despite similar levels of expression of protein measured by flow cytometry using an anti-MRGPRX2 antibody (*right* panel). The EC_50_ values were 160 ± 10 nM for WT and 283 ± 38 nM for N62S. In empty HEK293-Gα15 cells without MRGPRX2, no increase in calcium response was detected compared to either the MRGPRX2-WT or the MRGPRX2-N62S cells (data not shown). Thus, we confirmed that this unique assay can be applied to the HTS for detecting selective MRGPRX2 agonists.

### Primary *in vitro* HTS discovered MRGPRX2-selective hit compounds

GPCR HTS has enabled researchers to identify new and repurpose existing drugs and deorphanize GPCRs with unknown ligands (21). While several previous studies have identified some MRGPRX2 ligands (6), we performed an experimental HTS to expand on these studies and focus on FDA-approved drugs used as therapeutics for a wide variety of diseases (15). We designed an *in vitro* cell-based assay to assess MRGPRX2-dependent increase in intracellular calcium levels induced by MRGPRX2 ligands (**Figure 2A**).

However, genetic variation occurs in functional sites, potentially can alter drug responses. We predicted that MRGPRX2 LOF mutations seen in disease would render cells less reactive regarding calcium release induced by specific MRGPRX2 ligands. Thus, we performed the primary *in vitro* HTS employing HEK293-Gα15 cells stably expressing either the wild-type or the mutant receptors targeting N62S. This HTS allowed us to determine the activation of MRGPRX2 in a powerful, paired fashion (**Figure 2A**).

In the HTS, we determined MRGPRX2 activation by a Ca^2+^ mobilization assay that recorded a fluorescence change by utilizing a Functional Drug Screening System (FDSS 6000; Hamamatsu); we evaluated the compound effect using the calculated fluorescence ratio. We further applied the B-score normalization, which provided robust corrections for variation in fluorescence ratio (22). If a compound demonstrated high degrees of potency (caused more than three times the SD of the B-score of the library compounds), it was considered active as a hit compound. In MRGPRX2-WT cells, we identified 84 hit compounds (B-score value > 3*SD = 18.05) from 3,456 drugs for an overall hit rate of 3.04%. These agonists represented diverse drug classes according to their corresponding primary drug indications (**Supplementary Table 1**). Further, 34 out of 84 hit compounds were represented by five major drug classes (**Table 1**; **Figure 2B**): fluoroquinolone antibiotics (n=5, 15%); non-fluoroquinolone antibiotics (n=3, 9%); antidepressive/antipsychotic agents (n=8, 23%); anti-allergic agents (n=13, 38%) and antineoplastic agents (n=5, 15%). As expected, the fluoroquinolones displayed a high level of potency (B-score value: 31.71 ± 10.27). In contrast, decreased calcium influx was observed in MRGPRX2-MUT cells for all 34 hit compounds (percentages of reduction compared to MRGPRX2-WT cells: -89.01 ± 16.08), suggesting these ligands had relatively high selectivity for MRGPRX2.

**Table 1:** We identified 34 hit compounds (B-score ≥ 3*standard deviation) from a biological screening of the Hopkins FDA drug library utilizing HEK293-Gα15 cells stably expressing the wild-type MRGPRX2 receptor (MRGPRX2-WT).

**No.**	**Drug Name**	**PubChem CID**	**Primary Drug Indication**	**Approval Status**	**B-scores (WT)**	**B-scores (MUT)**	**Change of B-scores (%)**
1	Difloxacin Hydrochloride	56205	Antibiotics (Fluoroquinolone)	INN	44.81	-1.54	-103.45
2	Levofloxacin	149096	Antibiotics (Fluoroquinolone)	FDA	39.73	-0.74	-101.85
3	Norfloxacin	4539	Antibiotics (Fluoroquinolone)	FDA	28.07	0.05	-99.81
4	Clinafoxacin Hydrochloride	60062	Antibiotics (Fluoroquinolone)	USAN, INN	26.08	0.92	-96.48
5	Rufloxacin Hydrochloride	176015	Antibiotics (Fluoroquinolone)	INN, BAN	19.85	-0.15	-100.76
6	Alexidine Hydrochloride	102678	Antibiotics (Non-Fluoroquinolone)	USAN, INN	30.77	1.68	-94.54
7	Bacitracin A	10909430	Antibiotics (Non-Fluoroquinolone)	FDA	20.22	-0.17	-100.82
8	Rifampin	135398735	Antibiotics (Antitubercular)	FDA	23.94	0.37	-98.45
9	Fluoxetine	3386	Antidepressive Agents (Second-Generation)	FDA	36.30	7.14	-80.32
10	Imipramine Hydrochloride	8228	Antidepressive Agents (Tricyclic)	FDA	32.17	16.90	-47.47
11	Clomipramine Hydrochloride	68539	Antidepressive Agents (Tricyclic)	FDA	30.29	2.17	-92.83
12	Dibenzepin	9419	Antidepressive Agents (Tricyclic)	INN, BAN	25.48	-1.62	-106.37
13	Protriptyline Hydrochloride	4976	Antidepressive Agents (Tricyclic)	FDA	24.43	3.59	-85.30
14	Trimipramine Maleate	5282318	Antidepressive Agents (Tricyclic)	FDA	23.09	3.48	-84.91
15	Promazine Hydrochloride	5887	Antipsychotic Agents	FDA	23.47	8.13	-65.37
16	Thiothixene	941651	Antipsychotic Agents	FDA	20.36	5.11	-74.90
17	Cyproheptadine Hydrochloride	13770	Anti-Allergic Agents (Histamine H1 Antagonist)	FDA	41.66	2.29	-94.50
18	Brompheniramine Maleate	5281067	Anti-Allergic Agents (Histamine H1 Antagonist)	FDA	41.41	1.21	-97.07
19	Carbinoxamine Maleate	5282409	Anti-Allergic Agents (Histamine H1 Antagonist)	FDA	38.93	1.49	-96.18
20	Azelastine Hydrochloride	54360	Anti-Allergic Agents (Histamine H1 Antagonist)	FDA	36.21	3.47	-90.42
21	Thonzylamine Hydrochloride	6136	Anti-Allergic Agents (Histamine H1 Antagonist)	FDA	33.23	1.69	-94.91
22	Mebhydrolin Naphthalenesulfonate	5702169	Anti-Allergic Agents (Histamine H1 Antagonist)	INN, BAN, MI, JAN	25.34	1.93	-92.39
23	Dexchlorpheniramine Maleate	5281070	Anti-Allergic Agents (Histamine H1 Antagonist)	FDA	24.82	0.56	-97.76
24	Ketotifen Fumarate	5282408	Anti-Allergic Agents (Histamine H1 Antagonist)	FDA	23.14	0.81	-96.52
25	Loratadine	3957	Anti-Allergic Agents (Histamine H1 Antagonist)	FDA	21.95	-3.30	-115.04
26	Triprolidine Hydrochloride	5702129	Anti-Allergic Agents (Histamine H1 Antagonist)	FDA	20.71	0.68	-96.71
27	Ketotifen	3827	Anti-Allergic Agents (Histamine H1 Antagonist)	FDA	20.12	1.38	-93.13
28	Desloratidine	124087	Anti-Allergic Agents (Histamine H1 Antagonist)	FDA	19.79	0.29	-98.52
29	Brompheniramine	6834	Anti-Allergic Agents (Histamine H1 Antagonist)	FDA	18.22	0.64	-96.48
30	Masitinib	10074640	Antineoplastic Agents	INN	27.71	3.82	-86.20
31	Crizotinib	11626560	Antineoplastic Agents	FDA	22.06	-0.44	-101.99
32	Buserelin	50225	Antineoplastic Agents	FDA	20.46	5.73	-71.99
33	Danusertib	11442891	Antineoplastic Agents	INN	20.37	0.61	-97.00
34	Cepharanthine	10206	Antineoplastic Agents	JAN	19.73	1.14	-94.20

These hit compounds represent five major drug classes according to the MeSH Pharmacological Classification available in PubChem. We listed these hit compounds by general information (generic name, PubChem ID and approval status) and sorted by categories of primary drug indications as well as the B-score values in WT cells (in descending order within each class). The B-score values in HEK293-Gα15 cells stably expressing the MRGPRX2 N62S mutation (MRGPRX2-MUT) and the percentage of decrease in comparison to the MRGPRX2-WT cells were also presented.

BAN, British Approved Names; FDA, United States Food and Drug Administration; INN, International Nonproprietary Names; JAN, Japanese Adopted Names; MI, Merck Index; USAN, United States Accepted Names.

As a proof of concept, three out of five drug classes we discovered were reported previously displaying agonistic activity for MRGPRX2: fluoroquinolones sharing the tetrahydroisoquinoline or THIQ motif (6); antidepressive/antipsychotic agents and anti-allergic agents sharing the cationic amphiphilic characteristics (12). The largest drug class comprised 13 anti-allergic agents or antihistamines: these agents were mainly alkylamines (n=4, all were first-generation) and piperidines (n=5, two out of five were second-generation: loratadine and ketotifen), suggesting that both the first- and second-generation H1-antihistamines induced calcium-mobilization in MRGPRX2-WT cells, possibly *via* shared essential structural elements.

We also discovered another two drug classes potentially representing novel agonists for MRGPRX2: non-fluoroquinolone antibiotics including bacitracin A and antitubercular antibiotic rifampin as well as antineoplastic agents (e.g., masitinib). Hypersensitivity reactions have been reported for both bacitracin A and rifampin (23). However, diagnosis of rifampin hypersensitivity (ranging from pruritic skin eruptions to anaphylaxis) can be difficult because many reactions are likely not IgE-mediated. It is critical to confirm that these novel ligands can induce MRGPRX2-dependent activation of MCs, therefore may have clinical implications for diagnosis and prevention of DHRs.

Of note, as part of the validation process, we performed a separate screening of the same drug library utilizing HEK293-Gα15 cells without MRGPRX2, which served as the negative control for the primary screening. We found none of the MRGPRX2 agonists we identified (**Table 1**) showed elevated calcium release except for the anti-depressive/antipsychotic agents. Anti-depressive/antipsychotic agents are known to interact with other GPCRs such as histamine/serotonin/dopamine or adrenergic receptors. Thus, it is possible that these agents could activate other GPCRs, leading to altered (e.g., increased) sensitive of HEK293-Gα15 cells in responding to these drugs *via* the calcium release assay.

### Clustering analysis identified drug classes sharing high structural similarity

**Figure 3:**
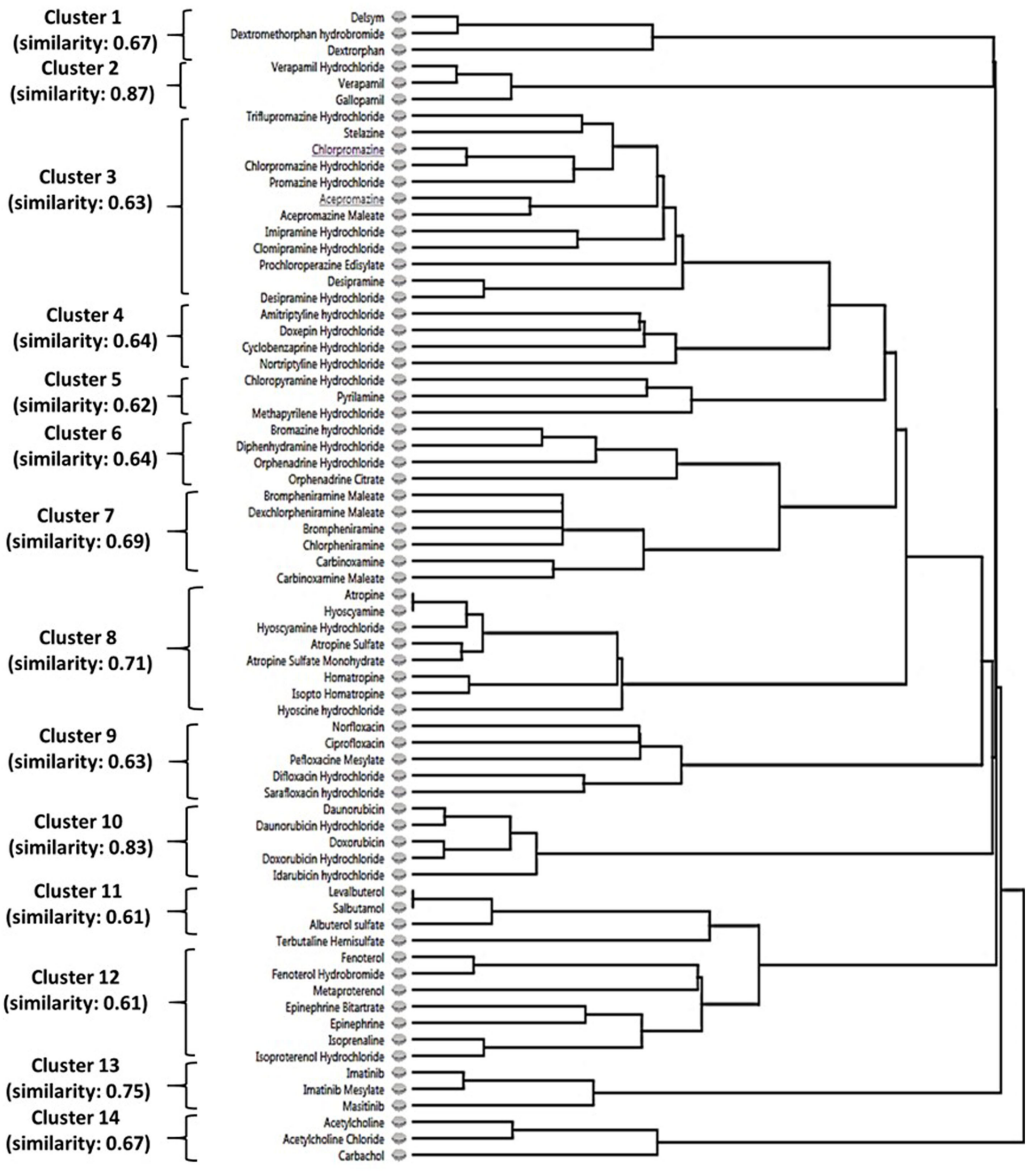
The structure-activity relationship of identified hit compounds for MRGPRX2. Fourteen clusters containing 70 MRGPRX2-selective hit compounds were displayed according to structural similarity using Forge. Criteria: Morgan=ECFP4; similarity cut-off=0.6.

Considering the effects of N62S mutation on the selectivity of hit compounds for MRGPRX2 activation, we further examined the differential responses between MRGPRX2-WT and MRGPRX2-MUT cells. We identified 226 hit compounds from 3,456 drugs displaying the largest differences of B-score between the two cell types by applying a more relaxed criterion: we defined positive hit as having a B-score difference greater than the mean+SD = 6.45 (n=189) or less than the mean-SD = -5.13 (n=37). Further, we explored the structure-activity relationship shared by these hit compounds using Forge, a molecular tool that can align structurally diverse compounds and provide a similarity score between the result molecule and the target molecule (18). Utilizing the 226 hits as an input, we observed 14 major clusters (with more than three members in each cluster) comprised 70 drug compounds sharing high structural similarity (criteria used: Morgan=ECFP4; cut-off=0.6. **Figure 3**).

As demonstrated in **Supplementary Table 2**, these 14 clusters represented diverse drug classes based on the primary drug indication and/or chemical structure: antitussive agents (opiate derivatives, n=3), calcium channel blockers (n=3), antipsychotic agents (phenothiazine tricyclic)/antidepressive agents, tricyclic (n=16), anti-allergic agents (n=13), mydriatics (anticholinergics, n=8), antibiotics (fluoroquinolones, n=5), antineoplastic antibiotics (anthracyclines, n=5), bronchodilator agents (n=11), antineoplastic agents (TKIs, n=3), and cholinergic receptor agonists (n=3). Thus, we confirmed the SAR among MRGPRX2 ligands sharing distinct chemical motifs. Further, most of these drug classes displayed decreased Ca^2+^ mobilization *via* the N62S mutation at a single concentration of 10 µM. However, these properties need to be validated by comparing the EC_50_ values between the two cell types.

### Antineoplastic agents induced calcium-mobilization

Our clustering analysis showed that various chemotherapeutic agents induced calcium-mobilization. Patients receiving multiple doses of chemotherapy can become sensitized to the drugs; subsequent exposure to these agents can lead to DHRs and death (24). Several protein tyrosine-kinase inhibitors (TKIs) provoked Ca^2+^ responses in our study. Masitinib is an orally available TKI of c-kit; it also inhibits PDGF and FGF receptors and fyn and lyn kinases (25). Masitinib interferes with the survival, migration, and activity of mast cells. In this role, masitinib has attracted attention for the treatment of mast cell tumors (MCTs), neuroinflammatory disorders, and neurodegenerative disorders. Currently, masitinib has only been approved for the treatment of canine MCTs (26). Imatinib is the first TKI introduced in 2001 to treat many leukemias, systemic mastocytosis, hypereosinophilic syndrome, dermatofibrosarcoma protuberans, and gastrointestinal stromal tumors (27). Our study provided evidence that TKIs could be potential agonists for MRGPRX2 and associated DHRs.

Anthracyclines such as daunorubicin (DNR, the first anthracycline) and doxorubicin (DOX) are widely used in human cancer chemotherapy (27, 28). However, their use is limited by cardiotoxicity and treatment resistance. The specific anthracyclines are distinguished by minor chemical changes that profoundly influence their half-lives, targetable tumor types, and toxicities. DOX is derived from DNR with the addition of a hydroxyl group on the carbon 14. The five anthracyclines displayed varying potencies in MRGPRX2-WT and MRGPRX2-MUT cells. **Supplementary Figure 1** shows the concentration-response curves for DNR (**panel A**), DNR hydrochloride (**panel B**), and DOX hydrochloride (**panel C**), with their corresponding EC_50_ values for MRGPRX2-WT cells (1.55, 0.051 and 0.131 μM, respectively). DNR hydrochloride also demonstrated the LOF property by EC_50_ values, comparing MRGPRX2-MUT to the MRGPRX2-WT cells (*P*=0.013). Desensitization and pre-treatment have been used to manage patients with DHRs to doxorubicin (27).

### Noval agonists elicited MRGPRX2-dependent degranulation of human mast cells

**Figure 4:**
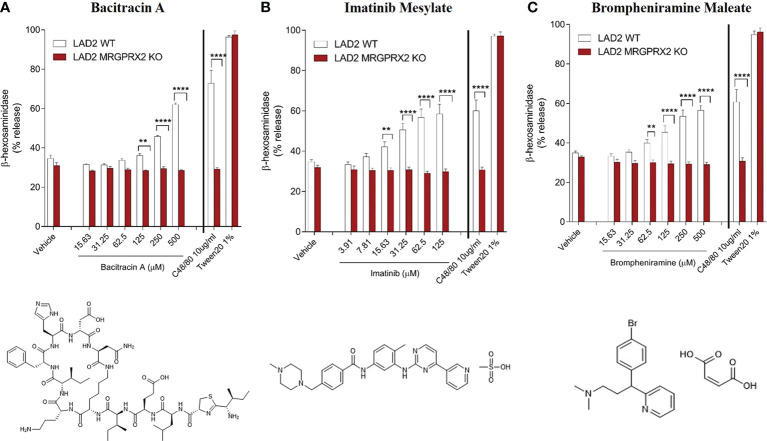
MRGPRX2-dependent degranulation of human mast cells (LAD2) measured by β-hexosaminidase release following stimulation by agonistic drugs bacitracin A **(A)**, imatinib mesylate **(B)** and brompheniramine maleate **(C)**. Significantly decreased degranulation was observed in LAD2 cells lacking MRGPRX2 for all three agonistic drugs (concentration as indicated), C48/80 (10 μg/mL) and Tween 20 (1%) were used as positive controls (*P*<0.0001, n=6). Two-way ANOVA with adjusting for multiple comparisons was performed for statistical analysis. Bars represent means ± SEMs. ****P <0.0001; **P < 0.01.

We further investigated whether these newly discovered candidate drugs can serve as agonists for MRGPRX2-dependent activation of MCs. We tested three drugs representing the TKIs (imatinib mesylate), non-fluoroquinolone antibiotics (bacitracin A) and antihistaminic agents (brompheniramine maleate) by using the human mast cell line LAD2 that express MRGPRX2 endogenously; MRGPRX2-deficient LAD2 cells was utilized as a negative control to confirm MRGPRX2-dependent degranulation of MCs. In LAD2 cells, we observed concentration-dependent degranulation measured by β-hexosaminidase release following stimulation by all three agonists (concentration as indicated) or C48/80 (10 μg/mL) as a positive control (**Figure 4**). In LAD2 cells lacking MRGPRX2, degranulation was decreased to the level of vehicle controls (two-way ANOVA, *P*< 0.0001). Specifically, the three drugs displayed varying potencies (from low to high) in terms of β-hexosaminidase release in LAD2 cells: 56.43% (brompheniramine maleate) and 61.94% (bacitracin A) at 500 μM, and 58.42% at 125 μM (imatinib mesylate). Our findings suggest that MRGPRX2 is crucial for hypersensitivity reactions *via* MC degranulation following stimulation with these agonistic drugs.

## Discussion

Pseudo-allergic DHRs are non-IgE-mediated hypersensitivity reactions elicited by an initial dose of medication and cause MC degranulation followed by the release of inflammatory and pro-inflammatory mediators (13). The clinical manifestation is similar to IgE-mediated allergic reactions, such as localization of symptoms to the inflamed tissue (*e.g.*, skin) or anaphylactic reactions (acute urticaria, anaphylaxis, bronchospasm, asthma) (29, 30). The majority of pseudo-allergic DHRs are mild (acute urticaria), but some cause anaphylaxis and can even be lethal, sometimes at the first encounter with the drug (often within minutes or hours), as no sensitization/prior exposure is required. Pseudo-allergic DHRs are still not predictable; thus, systematic studies on the causes and mechanisms are needed to understand such an off-target activity better. The present study builds on past findings of the mechanisms of drug-induced MC degranulation *via* MRGPRX2 and could facilitate the discovery of diverse MRGPRX2 agonists and personalized treatment strategies to mitigate pseudo-allergic DHRs.

Mutation (*e.g.*, single nucleotide polymorphism or SNP) in the MRGPRX2 gene may change its ligand-binding properties and therefore modulate the risk of anaphylactoid events. Several SNPs have been reported to abolish MC-mediated degranulation in response to MRGPRX2 ligands (13). These include four very rare (MAF< 0.01) missense SNPs (Gly165Glu, Asp184His, Trp243Arg, His259Tyr). For the two common missense SNPs, ligand-binding activity is unchanged for Asn16His. However, the Asn62Ser (N62S) mutation displays the LOF property associated with protection for chronic inflammatory disease (14). We confirmed the LOF function of the N62S mutation by comparing calcium mobilization in response to SP (a known ligand for MRGPRX2) and a novel ligand daunorubicin hydrochloride (belongs to a group of anthracyclines) between HEK293-Gα15 cells expressing wild-type and mutant receptors. The N62S mutation is thus a functional variant and good candidate for pharmacological studies targeting MRGPRX2 and pharmacogenetic studies in relevant disease cohorts.

Our HTS screening discovered several novel classes of agonists for MRGPRX2 commonly used as therapeutics for a wide variety of diseases (**Table 1**). Interestingly, we found that five antineoplastic drugs (*e.g.*, masitinib) shared a similar level of potency in activating the receptor (B-score value: 22.06 ± 3.27), in cells transfected with the wild-type receptor and at a single concentration of 10 µM. These findings prompted us to investigate the SARs that could be critical for receptor activation. Unlike other GPCRs with selective and limited agonists, MRGPRX2 is a low-affinity and low-selectivity receptor that can interact with diverse ligands. Based on our preliminary findings, we hypothesized that a common chemical motif of the agonists affects their capacities for MRGPRX2 activation. In a recent study, a group of cationic amphiphilic antidepressant drugs (clomipramine, paroxetine and desipramine) has shown MRGPRX2 agonistic activity associated with scratching behavior in mice and hypersensitivity reactions in human MCs (12). Further SAR experiments may aid in explaining drug-induced pruritus by similar cationic drugs. Indeed, the two largest clusters (#3 and #4) demonstrated MRGPRX2 agonistic activities in our study were sharing the tricyclic motif. These included tricyclic antidepressants (TCAs, n=8) and phenothiazines (n=8), a class of antipsychotics. Five main classifications of TCAs are commonly prescribed to treat depression, including the first-generation tricyclics and second-generation antidepressants: selective serotonin re-uptake inhibitors (SSRIs) and serotonin-norepinephrine re-uptake inhibitors (SNRIs). We discovered that the commonly used SSRIs (*e.g.*, fluoxetine) and SNRIs (*e.g.*, clomipramine and protriptyline) appeared to be robust agonists for MRGPRX2, along with several first-generation tricyclics (**Table 1**). TCAs are structurally similar to phenothiazines. The clustering trees showed that only the first-generation tricyclics and related agents were highly structurally similar with phenothiazine tricyclic antipsychotics.

Six distinct chemical classes exist for H1-antihistamines. Most H1-antagonists contain substituents in the aryl moieties (usually benzene), which influence antihistamine affinity, potency, and biodisposition (31). Our study clustered 13 first-generation H1-antihistamines according to the three major chemical classes (alkylamine, ethylenediamine, and ethanolamine); alkylamines displayed relatively higher potency. H1-antihistamines (more than 45 are available worldwide) comprise the largest class of medications used to treat allergic diseases (32). Second-generation H1-antihistamines (*e.g.*, desloratadine and loratadine), which are structurally similar to first-generation agents but more specific in action (selectively bind to peripheral histamine receptors), are the current medications of choice in patients with allergic rhinitis, allergic conjunctivitis, and chronic urticaria. In contrast, orally administered first-generation H1-antihistamines (antagonize H-1 receptors in the central nervous system) are no longer medications of choice due to adverse effects. Antihistamines are presumed to be able to lead to an anaphylaxis (33) and hypersensitivity reaction (34) in very rare cases. However, the observed effects were speculated as a continuation of the initial allergic or anaphylactic event (12). In our study, the first-generation H1-antihistamine brompheniramine maleate (alkylamine) displayed significantly higher potency for degranulation in LAD2 cells compared to cells lacking MRGPRX2 (**Figure 4**), suggesting it can provoke hypersensitivity reactions in MCs through a MRGPRX2-dependent manner.

Three-dimensional pharmacophore modelling confirmed that cationic amphiphilic drugs are potent agonists for MRGPRX2 (12). The structure of the MRGPRX2 complex has distinctive features for ligand-binding and G-protein coupling (35, 36). The shallow nature of the MRGPRX2 ligand-binding pocket enables easy recognition of structurally distinct cationic allergens by MRGPRX2 (36). In line with these discoveries, we found that a structurally heterogeneous group of drugs could potentially bind MRGPRX2, affecting its biologic activity. This group of drugs showed broad cross-reactivity among drugs of interest. The α5-helix of Gq engages the cytoplasmic core of MRGPRX2 (35). A crucial step in regulating downstream signalling events consists of GPCRs coupling to intracellular heterotrimeric G-proteins (37). Although the N62S mutation is located in the receptor’s cytoplasmic domain, it remains uncertain whether the mutation changes G-protein coupling through interaction with the α5-helix.

The most intriguing finding of our study is the discovery of antineoplastic agents as potent agonists for MRGPRX2. We have validated hit compounds sharing distinct structural similarities in two drug categories: anthracyclines and TKIs, as discussed further below. Anthracyclines possess a common structure that consists of a tetracyclic ring with quinone-hydroquinone groups linked to daunosamine by a glycosidic bond (28). DOX has largely replaced DNR for anticancer therapy. The main difference between DNR and its analog DOX is the presence of a hydroxyl group on the carbon 14 of DOX; this may account for its broad-spectrum action and a better efficiency against tumors. In our study, all three anthracyclines provoked a concentration-dependent activation of MRGPRX2-WT cells (**Supplementary Figure 1****)**. Moreover, the N62S LOF mutation reduced the activity of DNR hydrochloride, suggesting that carrier status of causal mutations of MRGPRX2 (*i.e.*, those affecting receptor biological function) can modify individual’s risk to anthracycline-induced DHRs. Testing for the presence of the N62S mutation could help predict individual response to anthracyclines and other drug classes.

Another group of chemotherapy agents, TKIs, also shared distinct structural similarity and functional properties. We confirmed that imatinib mesylate induced MRGPRX2-dependent MC degranulation (**Figure 4B**). Hypersensitivity reactions to imatinib include swelling, urticaria, acute generalized exanthematous pustulosis, exfoliative dermatitis, and Stevens-Johnson syndrome (38). Oral desensitization to imatinib have been attempted for a limited number of cases in the absence of an equivalent therapeutic option (39–42). Our findings may have relevance to TKI-induced DHRs mediated by MRGPRX2, future studies are warranted to characterize other TKIs such as crizotinib, which is used to treat metastatic non-small cell lung cancer.

Bacitracin A is a cyclic polypeptide antibiotic (by inhibiting the cell wall synthesis of Gram-positive bacteria) used to treat skin and eye infections, prevent wound infections, and to treat pneumonia and empyema in infants. Numerous reports have found an association between bacitracin and allergic contact dermatitis (ACD) (43). Bacitracin can also cause systemic events, including urticaria, sweating, dyspnea, hypotension, and potentially life-threatening anaphylactic shock (44, 45). Our study is the first to report that bacitracin can elicit MRGPRX2-dependent activation of MCs (**Figure 4A**), suggesting the non-IgE mediated mechanisms may be involved in the immunomodulatory effects of bacitracin.

Of note, we observed calcium activation signals in MRGPRX2-WT cells provoked by bronchodilators - agonists for the beta-2 adrenergic receptor (a classic GPCR) sharing the phenylethylamine structure (**Supplementary Table 1**). However, considering the possible cross-reactivity with other GPCRs such as MRGPRs in the context of Ca^2+^ activation, additional studies are warranted in the future to understand the mechanism of bronchodilator-induced calcium signalling.

There are limitations of our study. First, the potential adverse reactions of the MRGPRX2 agonists discovered by our study could be different between drugs given orally and parenterally. Vancomycin is a tricyclic glycopeptide antibiotic that is used intravenously to treat various gram-positive cocci bacterial infections (27). The most common reaction caused by vancomycin is infusion reaction (previously called “red man syndrome”). It appears to be caused by infusion rate-dependent direct MC degranulation and MRGPRX2 has been implicated in vancomycin infusion reactions (9, 46). Vancomycin is a weak agonist with a calculated EC_50_ of 60 μg/ml (47). In our study, we only observed modest level of activation of MRGPRX2-WT cells in response to vancomycin (B score=16.36) at 10 μM concentration, right below the B score cut-off of 18.05 for being an active hit. Thus, interrogating the relationship between peak blood concentrations of MRGPRX2 agonists and EC_50_ values in immediate hypersensitivity reactions may help understand the role of MRGPRX2 in adverse events and to provide clear clinical diagnostic criteria. Second, it is quite likely that for MRGPRX2 interactions with small ligands to lead to anaphylaxis, multiple mechanisms might be at play (*e.g.* genetic variations and other ecological perturbation such as modification of dosing and infusion time). Therefore, further validation utilizing *in vivo* models may help elucidate the complex interaction of diverse groups of agonists with MRGPRX2. Third, many patients develop non-specific symptoms on multiple drugs and MRGPRX2 may be important independently in the pathogenesis of itch and chronic urticaria. Therefore, it would be important to confirm MRGPRX2-dependent hypersensitivity phenotype induced by novel agonists in relevant disease cohorts. Finally, elucidating the mechanism of action (MoA) for identified hit compounds towards MRGPRX2 target has benefits as this knowledge may help discover new biology. The MoA may include understanding of hit compounds’ action on the cell signaling system or processes that are impacted by the hit compound through its interaction with MRGPRX2. For validation of selected hit compounds, we have measured cellular responsiveness, *i.e.*, mast cell degranulation as determined by β-hexosaminidase release. We demonstrated MRGPRX2-dependent MC degranulation induced by selected hit compounds in LAD2 lines (**Figure 4**). In future studies, we will further explore the possible MoA of identified hit compounds towards MRGPRX2 target (*e.g.*, signaling events downstream of receptor activation).

Our study represents the first HTS of a comprehensive collection of FDA-approved molecular entities to assess the activation of MRGPRX2 receptors in the context of a significant MRGPRX2 mutation. Further validation of hit agonists in disease-relevant cells and tissues is warranted. For example, it would be desirable to establish the correlation between agonists eliciting skin response and the expression of MRGPRX2 in the skin and to investigate plausible candidate mutations. As a starting point, our findings provide a valuable resource for cataloguing selective and potent MRGPRX2 agonists with distinct structural similarities and evaluating the personalized risk of DHRs mediated by the receptor.

## Data availability statement

The original contributions presented in the study are included in the article/**Supplementary Material**. Further inquiries can be directed to the corresponding author.

## Author contributions

The project was conceived and planned by LG. Experiments were carried out by FY, NL, QP and LG. Statistical analysis was performed by FY, NL and LG. The original draft was written by LG. FY, NL, JS, SS, DM and XD discussed the results and critically reviewed and revised the manuscript. All authors contributed to the article and approved the submitted version.

## Funding

This work was supported through internal funding from the Johns Hopkins University (Catalyst Award to LG); the Johns Hopkins University School of Medicine as part of the Core Coins Program (to LG) and 2R37NS054791 (to XD). The sponsor had no role in study design; collection, analysis, or interpretation of data; writing of the report; or the decision to submit the paper for publication.

## Acknowledgments

The authors acknowledge assistance for performing the high-throughput screening (HTS) on the Johns Hopkins Drug Library (JHDL) from the Johns Hopkins University ChemBioCORE Facility.

## Conflict of interest

XD is a co- founder and a scientific advisory board member of Escient Pharmaceuticals, a company focused on developing small molecules targeting MRGPRs. XD is also collaborating with GlaxoSmithKline (GSK) to develop small molecules targeting MRGPRX2.

The remaining authors declare that the research was conducted in the absence of any commercial or financial relationships that could be construed as a potential conflict of interest.

## Publisher’s note

All claims expressed in this article are solely those of the authors and do not necessarily represent those of their affiliated organizations, or those of the publisher, the editors and the reviewers. Any product that may be evaluated in this article, or claim that may be made by its manufacturer, is not guaranteed or endorsed by the publisher.

## Abbreviations

DHR, drug hypersensitivity reaction; GPCR, G-protein coupled receptor; HTS, high-throughput screening; MAF, minor allele frequency; TKI, tyrosine-kinase inhibitor. DNR, Daunorubicin; DOX, doxorubicin.
