# Supplementary figures and images for: Biological screening of a unique drug library targeting MRGPRX2
Source: Front Immunol. 2022 Oct 21;13:997389. doi: 10.3389/fimmu.2022.997389 (PMC9635925; doi:10.3389/fimmu.2022.997389)

# Supplementary Figure 1

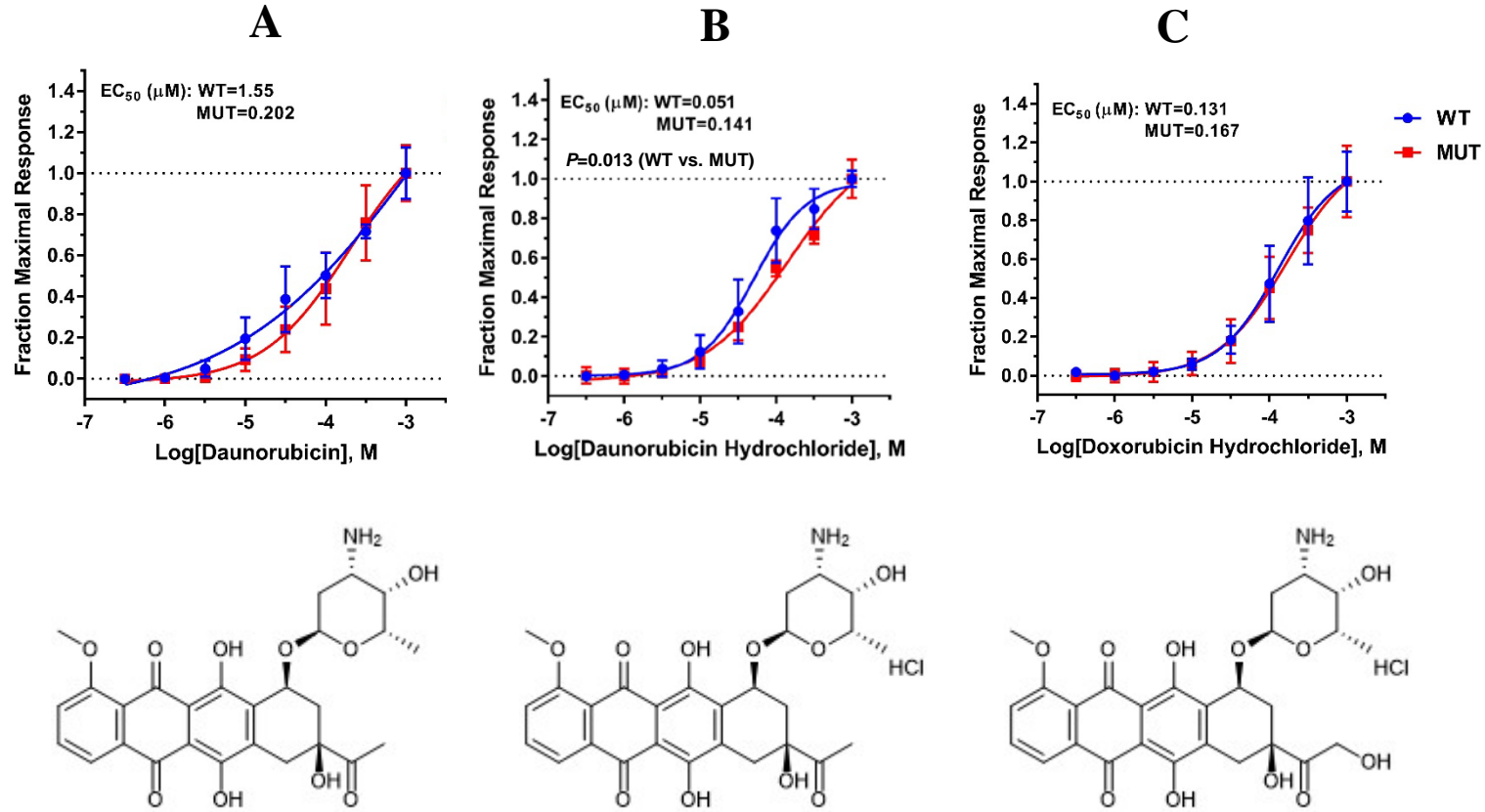

Supplement: Supplementary file 1 [file DataSheet_1.pdf]
