# Supplementary material for: Biological screening of a unique drug library targeting MRGPRX2
Source: Front Immunol. 2022 Oct 21;13:997389. doi: 10.3389/fimmu.2022.997389 (PMC9635925; doi:10.3389/fimmu.2022.997389)
Supplement: Supplementary file 2 [file Table_1.docx]

**Biological Screening of a Unique Drug Library Targeting MRGPRX2**

Fan Yang, MD, PhD^1,2†^; Nathachit Limjunyawong, PhD^3†^; Qi Peng, BS^3;^ John Schroeder, PhD^1^; Sarbjit Saini, MD^1^; Donald MacGlashan, MD, PhD^1^; Xinzhong Dong, PhD^3^; and Li Gao, MD, PhD^1*^

^1^Division of Allergy and Clinical Immunology, The Johns Hopkins University School of Medicine, Baltimore, MD; ^2^Department of Dermatology, Shengjing Hospital of China Medical University, Shenyang, China; ^3^The Solomon H. Snyder Department of Neuroscience, The Johns Hopkins University School of Medicine, Baltimore, MD

**Supplementary Materials and Methods**

***Generation of MRGPRX2 N62S mutant construct and sequence confirmation***

We performed site-directed mutagenesis to generate a mutant construct expressing the selected MRGPRX2 variant N62S (amino acid substitution from asparagine to serine at position 62). A mammalian expression construct of wild-type (WT) MRGPRX2 (MRGPRX2-WT) in pcDNA 3.1 vector was generated in Dr. Xinzhong Dong’s laboratory. We used the MRGPRX2-WT as backbones and the Q5® Site-Directed Mutagenesis Kit (New England Biolabs) to generate the mutant construct expressing the MRGPRX2 variant N62S (a single base pair substitution, from A to G). Constructs were sequence-confirmed (**Figure 1B**, *left* panel).

***Making the stable cell lines expressing MRGPRX2 WT and MUT constructs***

We cultured HEK293-Gα15 cells (HEK293 cells stably overexpressing G protein Gα15) on 24-well plates with a seeding density of 2.5x10^4^ cells/per well, cells were maintained at 37 ^o^C in a humidified atmosphere containing 5% CO_2_ 100 units/ml penicillin. After culture for 48 hours in Dulbecco’s Modified Eagle’s Medium (DMEM), pH 7.0 -7.6, supplemented with 10% FBS and 1% penicillin/streptomycin, we transiently transfected the wells with plasmids encoding the WT or mutant receptors utilizing Lipofectamine™ 3000 Reagent (Invitrogen). We selected clones stably expressing WT and mutant MRGPRX2 constructs by FACS sorting. To select the clones, we used a monoclonal antibody against MRGPRX2 (BioLegend, clones with relatively high expression of MRGPRX2 compared to unstained cells were selected). We followed the selection with a validation test of calcium release to MRGPRX2 ligands substance P (SP), at eight weeks after selection (**Figure 1B**, *middle* panel).

***Primary in vitro screen and selection of top candidate drugs***

We performed an experimental high-throughput screening (HTS) on the Johns Hopkins Drug Library (JHDL), which includes 1,811 (57%) FDA-approved drugs among 3,456 total compounds (28% of all known drugs worldwide). The remaining compounds in JHDL are catalogued in one or more of the resources below: British Approved Names (BAN); Dénominations Communes Françaises (DCF); International Nonproprietary Names (INN); Japanese Adopted Names (JAN); MI (Merck Index); National Formulary drug name (NF); and United States Accepted Names (USAN). The JHU drug library is stocked in 96 well plates at 10 mM in 100% DMSO and stored at -80^o^C. We used a final concentration of 10 uM for each compound and DMSO concentration of 0.1% (tested in triplicate). Columns 1 and 24 of each 384-well plate were used as in-plate controls and contained phosphate-buffered saline (PBS) with 1% DMSO (negative control) and 10 uM Substance P in PBS with 1% DMSO (positive control), respectively. We performed the HTS at the Johns Hopkins University ChemBioCORE Facility, and employed a HEK293-Gα15 cell line that stably expresses MRGPRX2 protein (both the wild-type (MRGPRX2-WT) and mutant (MRGPRX2-MUT) targeting the N62S variant (**Figure 2A**). We plated cells (MRGPRX2-WT and MRGPRX2-MUT) into 384-well plates (seeding density was 15,000 cells/well in 50 uL/well). The following day, we incubated cells with fluorescent dye-calcium 5 solution at 37 ^o^C (5% CO_2_) for 60 minutes and at room temperature for 30 minutes after removing the media. We added 10 uM compounds (each tested in triplicate) to the assay buffer with dye and recorded the change of fluorescence using a Hamamatsu FDSS 6000 kinetic imaging plate reader (Hamamatsu City, Japan). Fluorescence signals from both negative (0.1% DMSO) and positive (SP) wells were used for quality control of the assay. We evaluated compound effect using the calculated fluorescence ratio. We further calculated B-scores, which are a relative potency score similar to Z scores in that they are the ratio of an adjusted raw value in the numerator to a measure of variability in the denominator. B-scores provided an effective, non-controls-based methodology to deal with positional effects (Brideau C. *et al.*, J Biomol Screen. 2003). If the compound caused more than three times the standard deviation (SD) of the B-scores of the library compounds, the compound was considered active as an agonist of the MRGPRX2 protein (Zhang JH *et al.*, J Biomol Screen. 1999;4(2):67-73.).

***Validation of selected hit compounds and generation of dose-response curves***

As shown in Supplementary **Figure 1**, we selected anthracyclines (DNR-DOX), which are widely used in human cancer chemotherapy, for validation using the 384-well plate format. We applied eight concentrations on a logarithmic scale (-6.5, -6, -5.5, -5, -4.5, -4, -3.5, -3) with a maximum test concentration of 1 x 10^-3^ M or 1 mM to assess each drug in triplicate, and we repeated the experiment independently. We evaluated drug response by the calculated signal-to-background fluorescence ratio. We plotted dose-response curves over an eight concentration-effect range using GraphPad Prism (version 7), with the X-axis displaying the logarithm of concentration and the Y-axis displaying the relative response. Further, we calculated the EC_50_ value to determine the potency of each testing drug.

**Supplementary Results**

**Supplementary** **Table 1.** We identified 84 hit compounds (B-score ≥ 3*standard deviation) from a biological screening of the Hopkins FDA drug library utilizing HEK293-Gα15 cells stably expressing the wild-type MRGPRX2 receptor (MRGPRX2-WT). These hit compounds represent diverse drug classes according to the MeSH Pharmacological Classification available in PubChem. We listed these hit compounds by general information (generic name, PubChem ID and approval status) and sorted by categories of primary drug indications as well as the B-score values in WT cells (in descending order within each class). The B-score values in HEK293-Gα15 cells stably expressing the MRGPRX2 N62S mutation (MRGPRX2-MUT) and the percentage of decrease in comparison to the MRGPRX2-WT cells were also presented.

Abbreviations used: BAN, British Approved Names; DCF, Dénominations Communes Françaises; FDA, United States Food and Drug Administration; INN, International Nonproprietary Names; JAN, Japanese Adopted Names; MI, Merck Index; NF, National Formulary drug name; USAN, United States Accepted Names.

| **No.** | **Drug Name** | **PubChem CID** | **Primary Drug Indication** | **Approval Status** | **B-scores (WT)** | **B-scores (MUT)** | **Change of B-scores (%)** |
| --- | --- | --- | --- | --- | --- | --- | --- |
| 1 | Norepinephrine | 439260 | Adrenergic alpha-Agonists, Vasoconstrictor Agents | Y | 28.83 | 7.54 | -73.85 |
| 2 | Corticotropin A | N.A. | Adrenocorticotropic hormone (ACTH) | Y | 37.20 | 15.06 | -59.51 |
| 3 | Adenosine | 60961 | Anti-Arrhythmia Agents | Y | 35.61 | 0.32 | -99.09 |
| 4 | Difloxacin Hydrochloride | 56205 | Antibiotics (Fluoroquinolone) | INN | 44.81 | 1.54 | -103.45 |
| 5 | Levofloxacin | 149096 | Antibiotics (Fluoroquinolone) | Y | 39.73 | 0.74 | -101.85 |
| 6 | Norfloxacin | 4539 | Antibiotics (Fluoroquinolone) | Y | 28.07 | 0.05 | -99.81 |
| 7 | Clinafoxacin Hydrochloride | 60062 | Antibiotics (Fluoroquinolone) | USAN, INN | 26.08 | 0.92 | -96.48 |
| 8 | Rufloxacin Hydrochloride | 176015 | Antibiotics (Fluoroquinolone) | INN, BAN | 19.85 | 0.15 | -100.76 |
| 9 | Alexidine Hydrochloride | 102678 | Antibiotics (Non-Fluoroquinolone) | USAN, INN | 30.77 | 1.68 | -94.54 |
| 10 | Bacitracin A | 10909430 | Antibiotics (Non-Fluoroquinolone) | Y | 20.22 | 0.17 | -100.82 |
| 11 | Rifampin | 135398735 | Antibiotics (Antitubercular) | Y | 23.94 | 0.37 | -98.45 |
| 12 | Duloxetine Hydrochloride | 60834 | Antidepressive Agents, Second-Generation (SNRI) | Y | 44.22 | 0.64 | -98.55 |
| 13 | Fluoxetine | 3386 | Antidepressive Agents, Second-Generation (SSRI) | Y | 36.30 | 7.14 | -80.32 |
| 14 | Imipramine Hydrochloride | 8228 | Antidepressive Agents, Tricyclic | Y | 32.17 | 16.90 | -47.47 |
| 15 | Clomipramine Hydrochloride | 68539 | Antidepressive Agents, Tricyclic | Y | 30.29 | 2.17 | -92.83 |
| 16 | Dibenzepin | 9419 | Antidepressive Agents, Tricyclic | INN, BAN | 25.48 | 1.62 | -106.37 |
| 17 | Protriptyline Hydrochloride | 4976 | Antidepressive Agents, Tricyclic | Y | 24.43 | 3.59 | -85.30 |
| 18 | Trimipramine Maleate | 5282318 | Antidepressive Agents, Tricyclic | Y | 23.09 | 3.48 | -84.91 |
| 19 | Acepromazine Maleate | 6420038 | Antipsychotic Agents | USP, INN, BAN | 44.01 | 28.97 | -34.19 |
| 20 | Promazine Hydrochloride | 5887 | Antipsychotic Agents | Y | 23.47 | 8.13 | -65.37 |
| 21 | Thiothixene | 941651 | Antipsychotic Agents | Y | 20.36 | 5.11 | -74.90 |
| 22 | Trimethobenzamide Hydrochloride | 68385 | Antiemetics | Y | 18.56 | 0.14 | -99.27 |
| 23 | Caspofungin acetate | 6850808 | Antifungal Agents | Y | 29.98 | 16.90 | -43.63 |
| 24 | Pentamidine Isethionate | 8813 | Antifungal Agents, Antiprotozoal Agents (Trypanosoma, Leishmania) | Y | 37.48 | 0.43 | -98.84 |
| 25 | Pentamidine | 4735 | Antifungal Agents, Antiprotozoal Agents (Trypanosoma, Leishmania) | Y | 24.12 | 8.70 | -63.92 |
| 26 | Cyproheptadine Hydrochloride | 13770 | Antihistaminic Agents, Histamine H1 Antagonists | Y | 41.66 | 2.29 | -94.50 |
| 27 | Brompheniramine Maleate | 5281067 | Antihistaminic Agents, Histamine H1 Antagonists | Y | 41.41 | 1.21 | -97.07 |
| 28 | Carbinoxamine Maleate | 5282409 | Antihistaminic Agents, Histamine H1 Antagonists | Y | 38.93 | 1.49 | -96.18 |
| 29 | Azelastine Hydrochloride | 54360 | Antihistaminic Agents, Histamine H1 Antagonists | Y | 36.21 | 3.47 | -90.42 |
| 30 | Thonzylamine Hydrochloride | 6136 | Antihistaminic Agents, Histamine H1 Antagonists | Y | 33.23 | 1.69 | -94.91 |
| 31 | Mebhydrolin Naphthalenesulfonate | 5702169 | Antihistaminic Agents, Histamine H1 Antagonists | INN, BAN, MI, JAN | 25.34 | 1.93 | -92.39 |
| 32 | Dexchlorpheniramine Maleate | 5281070 | Antihistaminic Agents, Histamine H1 Antagonists | Y | 24.82 | 0.56 | -97.76 |
| 33 | Ketotifen Fumarate | 5282408 | Antihistaminic Agents, Histamine H1 Antagonists | Y | 23.14 | 0.81 | -96.52 |
| 34 | Loratadine | 3957 | Antihistaminic Agents, Histamine H1 Antagonists | Y | 21.95 | 3.30 | -115.04 |
| 35 | Triprolidine Hydrochloride | 5702129 | Antihistaminic Agents, Histamine H1 Antagonists | Y | 20.71 | 0.68 | -96.71 |
| 36 | Ketotifen | 3827 | Antihistaminic Agents, Histamine H1 Antagonists | Y | 20.12 | 1.38 | -93.13 |
| 37 | Desloratidine | 124087 | Antihistaminic Agents, Histamine H1 Antagonists | Y | 19.79 | 0.29 | -98.52 |
| 38 | Brompheniramine | 6834 | Antihistaminic Agents, Histamine H1 Antagonists | Y | 18.22 | 0.64 | -96.48 |
| 39 | Trimeprazine Tartrate | 441236 | Antihistaminic Agents, Histamine H1 Antagonists | Y | 27.11 | 19.11 | -29.51 |
| 40 | Amlodipine Besylate | 60496 | Antihypertensive Agents, Calcium Channel Blockers, Vasodilator Agents | Y | 31.57 | 3.87 | -87.76 |
| 41 | Diltiazem Hydrochloride | 62920 | Antihypertensive Agents, Calcium Channel Blockers, Vasodilator Agents | Y | 22.99 | 0.96 | -104.18 |
| 42 | Epoprostenol | 5282411 | Antihypertensive Agents | Y | 27.44 | 10.83 | -60.51 |
| 43 | Ramipril | 5362129 | Antihypertensive Agents, Angiotensin-Converting Enzyme Inhibitors | Y | 22.18 | 31.66 | 42.73 |
| 44 | Thimerosal | 16684434 | Anti-infective Agents; Preservatives, Pharmaceutical | Y | 18.55 | 0.33 | -98.25 |
| 45 | Almotriptan | 123606 | Antimigraine Agents, Serotonin Receptor Agonists | Y | 18.49 | 2.98 | -83.88 |
| 46 | Masitinib | 10074640 | Antineoplastic Agents | INN | 27.71 | 3.82 | -86.20 |
| 47 | Crizotinib | 11626560 | Antineoplastic Agents | Y | 22.06 | 0.44 | -101.99 |
| 48 | Buserelin | 50225 | Antineoplastic Agents | Y | 20.46 | 5.73 | -71.99 |
| 49 | Danusertib | 11442891 | Antineoplastic Agents | INN | 20.37 | 0.61 | -97.00 |
| 50 | Cepharanthine | 10206 | Antineoplastic Agents | JAN | 19.73 | 1.14 | -94.20 |
| 51 | Memantine Hydrochloride | 181458 | Antiparkinson Agents | Y | 26.34 | 0.05 | -99.82 |
| 52 | Benztropine Methanesulfonate | 3246155 | Antiparkinson Agents | Y | 26.25 | 2.81 | -89.29 |
| 53 | Benztropine | 1201549 | Antiparkinson Agents | Y | 21.42 | 1.35 | -93.68 |
| 54 | Orphenadrine Citrate | 83823 | Antiparkinson Agents; Muscle Relaxants, Central | Y | 19.77 | 0.60 | -96.96 |
| 55 | Dextromethorphan hydrobromide | 5464025 | Antitussive Agents | Y | 38.47 | 84.34 | 119.23 |
| 56 | Dextromethorphan Hydrobromide Monohydrate | 5462351 | Antitussive Agents | Y | 23.02 | 54.88 | 138.39 |
| 57 | Pamidronate disodium | 73351 | Bone Density Conservation Agents | Y | 30.08 | 18.72 | -37.79 |
| 58 | Formoterol | 3410 | Bronchodilator Agents | Y | 46.02 | 17.88 | -61.14 |
| 59 | Isoetharine Mesylate | 23702 | Bronchodilator Agents | Y | 29.54 | 0.60 | -97.97 |
| 60 | Epinephrine Bitartrate | 5815 | Bronchodilator Agents | Y | 25.60 | 11.72 | -54.24 |
| 61 | Fenoterol | 3343 | Bronchodilator Agents | USAN, INN, BAN, JAN | 23.48 | 13.78 | -41.33 |
| 62 | Colforsin | 47936 | Bronchodilator Agents | INN | 21.10 | 0.76 | -96.40 |
| 63 | Isoproterenol Hydrochloride | 5807 | Bronchodilator Agents | Y | 20.95 | 0.05 | -100.26 |
| 64 | Metaproterenol | 4086 | Bronchodilator Agents | Y | 20.54 | 2.41 | -88.28 |
| 65 | Fenoterol Hydrobromide | 5702161 | Bronchodilator Agents | USAN, INN, BAN, JAN | 20.31 | 0.03 | -100.16 |
| 66 | Isoproterenol | 3779 | Bronchodilator Agents | Y | 19.12 | 6.75 | -64.69 |
| 67 | Isoprenaline hydrochloride | 5807 | Bronchodilator Agents, Cardiotonic Agents | Y | 21.82 | 0.05 | -100.25 |
| 68 | Carbachol | 5831 | Cholinergic Agonists | Y | 42.55 | 63.57 | 49.41 |
| 69 | Methacholine Chloride | 6114 | Cholinergic Agonists | Y | 26.04 | 37.31 | 43.29 |
| 70 | Acetylcholine | 187 | Cholinergic Agonists | Y | 23.36 | 80.61 | 245.01 |
| 71 | Bethanechol Chloride | 11548 | Cholinergic Agonists | Y | 19.27 | 37.55 | 94.89 |
| 72 | Arecoline Hydrobromide | 9301 | CNS stimulant | NF XII, MI | 28.78 | 58.08 | 101.79 |
| 73 | Acetylcholine Chloride | 6060 | Neurotransmitter | Experimental | 47.11 | 65.23 | 38.45 |
| 74 | Galantamine | 9651 | Cholinesterase Inhibitors | Y | 40.50 | 1.25 | -96.90 |
| 75 | Galantamine Hydrobromide | 121587 | Cholinesterase Inhibitors | Y | 19.90 | 1.33 | -93.34 |
| 76 | Atropine Sulfate | 60196398 | Mydriatics | Y | 41.43 | 0.13 | -100.31 |
| 77 | Homatropine Hydrobromide | 6419941 | Mydriatics | USP, JAN, BAN | 26.88 | 3.26 | -87.87 |
| 78 | Atropine Sulfate Monohydrate | 656678 | Mydriatics | Y | 24.16 | 0.90 | -96.27 |
| 79 | Atropine | 174174 | Mydriatics | Y | 18.59 | 0.95 | -105.08 |
| 80 | Iproheptine | 19917 | Nasal decongestant | JAN | 26.61 | 10.27 | -61.42 |
| 81 | Dinoprostone (Prostaglandin E2) | 5280360 | Oxytocics | Y | 40.59 | 17.30 | -57.38 |
| 82 | Pizotyline Malate | 168993 | Vascular headache | USAN, INN | 39.73 | 1.29 | -96.75 |
| 83 | Alprostadil | 5280723 | Vasodilator Agents | Y | 42.04 | 11.08 | -73.65 |
| 84 | Moxisylyte Hydrochloride | 6420039 | Vasodilator Agents | INN, BAN, DCF, MI, JAN | 23.69 | 1.32 | -94.42 |

**Supplementary** **Table 2.** Clustering of MRGPRX2-responsive top candidate drugs (n=70) screened from the Hopkins FDA drug library (displaying the largest differential responses between MRGPRX2-WT and MRGPRX2-MUT cells). We listed the 70 compounds in 14 clusters (at least three compounds in each cluster) by general information (generic name, PubChem ID, categories of substructure, and primary indications), B-score for each drug, each cell line, and the differences (percentage of B-score changes between cells stably expressing the MRGPRX2-WT and MRGPRX2-MUT cells, arranged by descending order), together with cluster number and similarity (score ≥ 0.6) for designated clusters according to Forge.

| **No.** | **Drug Name** | **PubChem CID** | **Drug Indication/Chemical Class** | **B-scores (WT)** | **B-scores (MUT)** | **Change of B-scores (%)** | **Cluster# (Forge)*** | **Cluster Similarity** |
| --- | --- | --- | --- | --- | --- | --- | --- | --- |
| 1 | Dextromethorphan (Delsym) | 5464025 | Antitussive Agents (opiate derivatives) | 38.47 | 84.34 | 119.23 | 1 | 0.67 |
| 2 | Dextrorphan | 5360697 | Antitussive Agents (opiate derivatives) | 31.94 | 68.33 | 113.94 | 1 | 0.67 |
| 3 | Dextromethorphan hydrobromide | 5462351 | Antitussive Agents (opiate derivatives) | 23.02 | 54.88 | 138.39 | 1 | 0.67 |
| 4 | Verapamil Hydrochloride | 62969 | Calcium Channel Blockers | 9.68 | 1.41 | -85.46 | 2 | 0.87 |
| 5 | Gallopamil | 1234 | Calcium Channel Blockers | 7.16 | 0.2 | -97.19 | 2 | 0.87 |
| 6 | Verapamil | 2520 | Calcium Channel Blockers | 5.44 | -1.44 | -126.44 | 2 | 0.87 |
| 7 | Acepromazine Maleate | 6420038 | Antipsychotic Agents (phenothiazine tricyclic) | 44.01 | 28.97 | -34.19 | 3 | 0.63 |
| 8 | Imipramine Hydrochloride | 8228 | Antidepressive Agents, Tricyclic | 32.17 | 16.9 | -47.47 | 3 | 0.63 |
| 9 | Clomipramine Hydrochloride | 68539 | Antipsychotic Agents (phenothiazine tricyclic) | 30.29 | 2.17 | -92.83 | 3 | 0.63 |
| 10 | Promazine Hydrochloride | 5887 | Antipsychotic Agents (phenothiazine tricyclic) | 23.47 | 8.13 | -65.37 | 3 | 0.63 |
| 11 | Acepromazine | 6077 | Antipsychotic Agents (phenothiazine tricyclic) | 22.99 | 43.26 | 88.18 | 3 | 0.63 |
| 12 | Desipramine | 2995 | Antidepressive Agents, Tricyclic | 13.96 | 1.19 | -91.45 | 3 | 0.63 |
| 13 | Chlorpromazine | 2726 | Antipsychotic Agents (phenothiazine tricyclic) | 10.2 | -0.05 | -100.54 | 3 | 0.63 |
| 14 | Triflupromazine Hydrochloride | 66069 | Antipsychotic Agents (phenothiazine tricyclic) | 8.62 | 0.85 | -90.15 | 3 | 0.63 |
| 15 | Chlorpromazine Hydrochloride | 6240 | Antipsychotic Agents (phenothiazine tricyclic) | 7.97 | 2.69 | -66.27 | 3 | 0.63 |
| 16 | Stelazine | 2913535 | Antipsychotic Agents (phenothiazine tricyclic) | 7.75 | 1.08 | -86.07 | 3 | 0.63 |
| 17 | Desipramine Hydrochloride | 65327 | Antidepressive Agents, Tricyclic | 7.48 | -2.96 | -139.62 | 3 | 0.63 |
| 18 | Prochloroperazine Edisylate | 91499 | Antipsychotic Agents (phenothiazine tricyclic) | 5.68 | 15.61 | 174.91 | 3 | 0.63 |
| 19 | Cyclobenzaprine Hydrochloride | 22576 | Antidepressive Agents, Tricyclic | 16.49 | 1.22 | -92.62 | 4 | 0.64 |
| 20 | Amitriptyline hydrochloride | 11065 | Antidepressive Agents, Tricyclic | 16.31 | 1.83 | -88.77 | 4 | 0.64 |
| 21 | Nortriptyline Hydrochloride | 441358 | Antidepressive Agents, Tricyclic | 16.29 | 2.78 | -82.96 | 4 | 0.64 |
| 22 | Doxepin Hydrochloride | 6419921 | Antidepressive Agents, Tricyclic | 12.95 | 1.85 | -85.71 | 4 | 0.64 |
| 23 | Chloropyramine Hydrochloride | 80311 | Antihistaminic Agents (ethylenediamine) | 14.6 | 3.24 | -77.83 | 5 | 0.62 |
| 24 | Pyrilamine | 4992 | Antihistaminic Agents (ethylenediamine) | 13.36 | 0.23 | -98.24 | 5 | 0.62 |
| 25 | Methapyrilene Hydrochloride | 8667 | Antihistaminic Agents (ethylenediamine) | 9.09 | -0.09 | -100.94 | 5 | 0.62 |
| 26 | Orphenadrine Citrate | 83823 | Antihistaminic Agents (ethanolamine) | 19.77 | 0.6 | -96.96 | 6 | 0.64 |
| 27 | Bromazine hydrochloride | 519514 | Antihistaminic Agents (ethanolamine) | 11.37 | -0.05 | -100.41 | 6 | 0.64 |
| 28 | Diphenhydramine Hydrochloride | 8980 | Antihistaminic Agents (ethanolamine) | 10.26 | 0.66 | -93.55 | 6 | 0.64 |
| 29 | Orphenadrine Hydrochloride | 9568 | Antihistaminic Agents (ethanolamine) | 7.68 | 0.58 | -92.44 | 6 | 0.64 |
| 30 | Brompheniramine Maleate | 5281067 | Antihistaminic Agents (alkylamine) | 41.41 | 1.21 | -97.07 | 7 | 0.69 |
| 31 | Carbinoxamine Maleate | 5282409 | Antihistaminic Agents (alkylamine) | 28.78 | 1.49 | -94.84 | 7 | 0.69 |
| 32 | Dexchlorpheniramine Maleate | 5281070 | Antihistaminic Agents (alkylamine) | 24.82 | 0.56 | -97.76 | 7 | 0.69 |
| 33 | Brompheniramine | 6834 | Antihistaminic Agents (alkylamine) | 18.22 | 0.64 | -96.48 | 7 | 0.69 |
| 34 | Chlorpheniramine | 2725 | Antihistaminic Agents (alkylamine) | 9.6 | 0.88 | -90.88 | 7 | 0.69 |
| 35 | Carbinoxamine | 2564 | Antihistaminic Agents (ethanolamine) | 11.13 | 0.12 | -98.93 | 7 | 0.69 |
| 36 | Atropine Sulfate | 60196398 | Mydriatics (Anticholinergic) | 41.43 | -0.13 | -100.31 | 8 | 0.71 |
| 37 | Isopto Homatropine | 5821 | Mydriatics (Anticholinergic) | 26.88 | 3.26 | -87.87 | 8 | 0.71 |
| 38 | Atropine Sulfate Monohydrate | 656678 | Mydriatics (Anticholinergic) | 24.16 | 0.9 | -96.27 | 8 | 0.71 |
| 39 | Atropine | 174174 | Mydriatics (Anticholinergic) | 18.59 | -0.95 | -105.08 | 8 | 0.71 |
| 40 | Hyoscyamine | 154417 | Mydriatics (Anticholinergic) | 17.72 | 0.65 | -96.31 | 8 | 0.71 |
| 41 | Homatropine | 5282593 | Mydriatics (Anticholinergic) | 17.51 | 0.41 | -97.68 | 8 | 0.71 |
| 42 | Hyoscyamine Hydrochloride | 12310717 | Mydriatics (Anticholinergic) | 11.54 | 0.69 | -94.01 | 8 | 0.71 |
| 43 | Hyoscine hydrochloride | 6852406 | Mydriatics (Anticholinergic) | 11.2 | 0.57 | -94.9 | 8 | 0.71 |
| 44 | Difloxacin Hydrochloride | 56205 | Antibiotics (Fluoroquinolone, THIQ) | 44.81 | -1.54 | -103.45 | 9 | 0.63 |
| 45 | Norfloxacin | 4539 | Antibiotics (Fluoroquinolone, THIQ) | 28.07 | 0.05 | -99.81 | 9 | 0.63 |
| 46 | Sarafloxacin hydrochloride | 56207 | Antibiotics (Fluoroquinolone, THIQ) | 15.97 | 0 | -100 | 9 | 0.63 |
| 47 | Pefloxacine Mesylate | 119525 | Antibiotics (Fluoroquinolone, THIQ) | 7.86 | -0.79 | -110.02 | 9 | 0.63 |
| 48 | Ciprofloxacin | 2764 | Antibiotics (Fluoroquinolone, THIQ) | 7.33 | 1.91 | -74 | 9 | 0.63 |
| 49 | Idarubicin hydrochloride | 636362 | Antineoplastic, Antibiotics (anthracycline) | 11.57 | 39.57 | 241.86 | 10 | 0.83 |
| 50 | Daunorubicin | 30323 | Antineoplastic, Antibiotics (anthracycline) | 8.85 | 37.13 | 319.41 | 10 | 0.83 |
| 51 | Doxorubicin | 31703 | Antineoplastic, Antibiotics (anthracycline) | 6.91 | 20.7 | 199.56 | 10 | 0.83 |
| 52 | Doxorubicin Hydrochloride | 443939 | Antineoplastic, Antibiotics (anthracycline) | 3.21 | 15.15 | 372.43 | 10 | 0.83 |
| 53 | Daunorubicin Hydrochloride | 62770 | Antineoplastic, Antibiotics (anthracycline) | 2.95 | 15.19 | 414.49 | 10 | 0.83 |
| 54 | Terbutaline Hemisulfate | 441334 | Bronchodilator Agents | 15.97 | 1.91 | -88.03 | 11 | 0.61 |
| 55 | Salbutamol | 2083 | Bronchodilator Agents | 13.61 | 0.36 | -97.38 | 11 | 0.61 |
| 56 | Levalbuterol | 123600 | Bronchodilator Agents | 12.18 | 1.63 | -86.62 | 11 | 0.61 |
| 57 | Albuterol sulfate | 39859 | Bronchodilator Agents | 12.09 | 2.96 | -75.51 | 11 | 0.61 |
| 58 | Epinephrine Bitartrate | 5815 | Bronchodilator Agents | 25.6 | 11.72 | -54.24 | 12 | 0.61 |
| 59 | Fenoterol | 3343 | Bronchodilator Agents | 23.48 | 13.78 | -41.33 | 12 | 0.61 |
| 60 | Isoproterenol Hydrochloride | 5807 | Bronchodilator Agents | 20.95 | -0.05 | -100.26 | 12 | 0.61 |
| 61 | Metaproterenol | 4086 | Bronchodilator Agents | 20.54 | 2.41 | -88.28 | 12 | 0.61 |
| 62 | Fenoterol Hydrobromide | 5702161 | Bronchodilator Agents | 20.31 | -0.03 | -100.16 | 12 | 0.61 |
| 63 | Isoprenaline | 3779 | Bronchodilator Agents | 19.12 | 6.75 | -64.69 | 12 | 0.61 |
| 64 | Epinephrine | 5816 | Bronchodilator Agents | 17.52 | 8.28 | -52.73 | 12 | 0.61 |
| 65 | Masitinib | 10074640 | Antineoplastic Agents (TKIs) | 27.71 | 3.82 | -86.2 | 13 | 0.75 |
| 66 | Imatinib Mesylate | 123596 | Antineoplastic Agents (TKIs) | 12.86 | -0.56 | -104.39 | 13 | 0.75 |
| 67 | Imatinib | 5291 | Antineoplastic Agents (TKIs) | 10.86 | -0.18 | -101.63 | 13 | 0.75 |
| 68 | Acetylcholine Chloride | 6060 | Cholinergic Agonists | 47.11 | 65.23 | 38.45 | 14 | 0.67 |
| 69 | Carbachol | 5831 | Cholinergic Agonists | 42.55 | 63.57 | 49.41 | 14 | 0.67 |
| 70 | Acetylcholine | 187 | Cholinergic Agonists | 23.36 | 80.61 | 245.01 | 14 | 0.67 |

**Supplementary Figure 1**. Dose-response curves over a full concentration-effect range of validated MRGPRX2-selective anthracyclines: (A) Daunorubicin (DNR), (B) DNR hydrochloride, and (C) Doxorubicin (DOX) hydrochloride. The X-axis depicts the logarithm of concentration, and the Y-axis depicts the relative response. In response to daunorubicin hydrochloride, cells expressing the MRGPRX2 N62S mutation demonstrated a *loss-of-function* (LOF) property by EC_50_ values compared to cells expressing the wild-type MRGPRX2 (*P*=0.013, unpaired t-test with adjustment for multiple comparisons).
